# Diagnostic yield of renal biopsies: a retrospective single center review

**DOI:** 10.1186/1471-2369-10-11

**Published:** 2009-05-21

**Authors:** Bari Scheckner, Alexandra Peyser, Jacob Rube, Freya Tarapore, Rachel Frank, Suzanne Vento, Cathy Hoffman, Elsa Valderrama, Douglas Charney, Beatrice Goilav, Howard Trachtman

**Affiliations:** 1 Department of Pediatrics, Division of Nephrology, Schneider Children's Hospital, New Hyde Park, New York of North Shore-LIJ Health System, Long Island Campus of the Albert Einstein College of Medicine, New York, USA; 2 Department of Pathology, Schneider Children's Hospital, New Hyde Park, New York of North Shore-LIJ Health System, Long Island Campus of the Albert Einstein College of Medicine, New York, USA

## Abstract

**Background:**

Previous studies have examined the spectrum of diseases identified with a kidney biopsy and the complications of the procedure. However, few studies have examined the utility of the test to clarify the diagnosis and guide treatment of pediatric patients. This retrospective, single-center chart review was performed to test the hypothesis that at least 80% of native kidney biopsies provide clinically valuable information that rationally guides diagnosis and patient management.

**Methods:**

200 biopsies performed between January 1, 2000 and June 30, 2008 were reviewed. A scheme composed of six categories was devised to classify the utility of each kidney biopsy.

**Results:**

196 complete case files were available for review. Twenty-four (12.2%) biopsies did not shed light on the diagnosis and were unhelpful in patient management – 21 biopsies (10.7%) were non-diagnostic and 3 (1.5%) failed to yield enough tissue for examination. The number of unhelpful biopsies did not cluster in any specific disease entity.

**Conclusion:**

Our findings provide guidance to nephrologists about the total risk of a kidney biopsy, including uninformative results, when seeking informed consent for the procedure. The results suggest an appropriate balance has been reached which maximizes the use of kidney biopsies while minimizing the risk of this invasive procedure (word count: 202).

## Background

A kidney biopsy is an important diagnostic procedure in nephrology and can aid in determining the appropriate diagnosis, treatment, and prognosis for specific patients. Based upon physical examination, urinalysis and blood tests, physicians attempt to make a clinical diagnosis which is used to guide treatment. This is not always feasible using non-invasive tools, and in such cases a kidney biopsy may need to be performed to confirm or determine the diagnosis, severity, and urgency of therapy.

A kidney biopsy may not always provide information that is needed or pertinent to the clinical scenario. Sometimes an adequate tissue sample of the kidneys cannot be obtained or the information provided from the biopsy fails to shed any light on the patients' symptoms or clinical data. Due to the invasiveness of the procedure, it is important, when recommending a biopsy for an individual patient, to bear in mind not only the risks of the procedure itself but the distinct possibility that it might prove unhelpful in the patient's management.

There are a number of recent studies that have detailed the array of diagnoses obtained from kidney biopsies in adults [1-8] and children [9-13] with renal disease. Other reports have assessed the clinical impact that a biopsy may have, but they focused primarily on the risk factors and resulting complications of performing renal biopsies [4]. In this study, we defined a novel system that enabled us to ascertain the usefulness of a particular biopsy by analyzing predictions of the diagnosis pre-biopsy and the physician's treatment decision post biopsy. Using this scheme, we tested the hypothesis that at least 80% of native kidney biopsies provided clinically valuable information that rationally guided the formulation of the patient's prognosis and treatment.

## Methods

### Patients

A database of biopsy procedures was obtained from the Department of Pathology that contained information on all native kidney biopsies completed at Schneider Children's Hospital between January 1, 2000 and June 31, 2008. The database included the patient's name, date of biopsy, date of birth, and final diagnosis.

The medical records of patients were retrieved from active clinical files or from off-site storage. The following data were extracted from each available chart: age, gender, blood pressure, dipstick urinalysis results, height (cm), serum creatinine, estimated glomerular filtration rate (GFRe), and urine protein/creatinine ratio in an early morning specimen at the last evaluation prior to the biopsy. In addition, the reason for biopsy, physician's predicted diagnosis, the biopsy-confirmed diagnosis, and treatment outcome were recorded. The predicted diagnosis was based on the correspondence from the nephrologist who performed the biopsy to the patient's primary physician or was based on the clinical diagnosis recorded on the biopsy report at the time of submission of the tissue sample.

Classification of diagnostic outcomes of the biopsy: A six-point scheme was designed to determine whether the biopsy served as a useful tool in establishing treatment. If the physician conclusively predicted a particular diagnosis which the biopsy confirmed, the patient was categorized as type 1. A type 2 categorization denoted a biopsy that confirmed one of multiple possible diagnoses. A type 3 categorization accounted for an incorrect pre-biopsy prediction but in which the biopsy finding was conclusive and sufficient to determine appropriate treatment. If the biopsy was used to determine the severity of an established disease, category 4 was assigned to that patient. Failures were identified as either content based or technical and were separated accordingly. Thus, patients in whom the biopsy was insufficient on its own to define prognosis and treatment were categorized as type 5. Technical failures, namely an inability to obtain tissue, were categorized as type 6.

Each case was independently reviewed by one of the four primary authors (AP, JR, BS, FT). Clinical and biopsy reports were recorded and categorized according to the scheme outlined above. Agreement between all pairs of primary reviewers was reached in over 85% of all cases. When there was a discrepancy between the primary reviewers, an independent observer (RF), who did not perform the biopsy, reviewed the case. This reviewer also checked randomly selected charts for accuracy and agreed with the primary reviewers in all cases. A nurse was chosen to adjudicate conflicts among the primary reviewers and to confirm accuracy of the chart reviews because this person would be less likely to be biased in interpreting the utility of the kidney biopsy procedures. The nephrologist who did the kidney biopsy did not participate in the categorization of the informational outcome of the procedure to avoid potential bias.

### Statistical Methods

Data are reported as mean ± SD. Differences in proportion were assessed by the chi square or Fisher exact test as appropriate. Differences were considered statistically significant if the P value was <0.05. All information was gathered on a pre-approved form.

Data were de-identified and coded by study number in accordance with the Health Insurance Portability and Accountability Act guidelines (HIPAA). This chart review was approved by the Institutional Review Board of North Shore-LIJ Health System.

## Results

A total of 200 biopsies were identified in the pathology database. All charts were retrieved. 4 charts were excluded because the biopsy was done at a different center and the diagnostic assessment and its impact on management by the attending physician could not be directly assessed. Thus, this report is based on the outcomes of 196 kidney biopsies.

**Figure 1:**
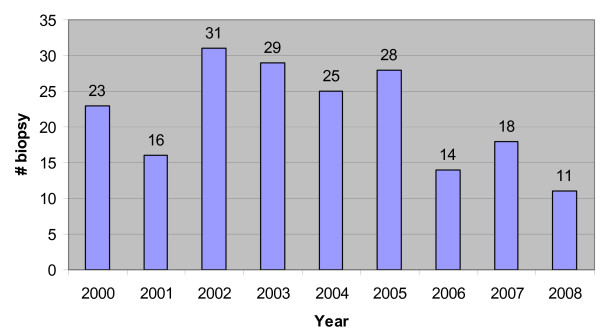
**This graph illustrates the annual number of biopsies during the study period, 01/2000 – 06/2008**.

The number of biopsies per year is illustrated in Figure 1 which demonstrates a fairly steady rate of performance of this procedure. The vast majority, 183 out of 196 (93%), of the procedures were performed in the outpatient setting. The hospitalized patients represented the cohort in which a kidney biopsy was done to assess acute renal dysfunction. The clinical characteristics of the patients are summarized in Table 1. Of note, 77 (59%) of the patients had U_P/C _>1 and 30 (15%) had GFRe <90 mL/min/1.73 m^2^.

**Table 1:** Kidney Biopsy Patient Clinical Features

GFRe	131.9 ± 65
**Urine protein/creatinine**	3.2 ± 4.6

**Systolic blood pressure**	117 ± 17

**Diastolic blood pressure**	70 ± 11

**Age (yr)**	12 ± 5

≤ 10 years	37%

Proteinuria*	77.36%

Hematuria**	86%

Data are provided as mean ± SD.

*Proteinuria indicates ≥ 1+ by dipstick testing; **Hematuria indicates ≥ small by dipstick testing.

Eleven of the patients who had biopsies underwent repeat biopsies. Nine patients had a second biopsy, one had three, and another underwent four procedures. In the last case, of the four biopsies performed, one of the biopsies was a technical failure. In two of these patients, the first biopsies were normal or non-diagnostic and subsequent biopsies showed FSGS and MPGN, respectively. In two other patients, neither the original nor follow up biopsies resulted in a definitive diagnosis. In the remaining 6 patients, the repeat kidney biopsy was done to assess response to treatment, 4 in patients with SLE and 2 in patients with MPGN.

**Figure 2:**
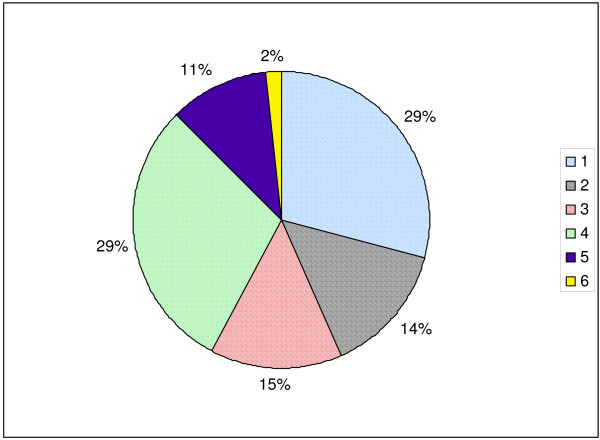
Category 1: Predicted diagnosis confirmed Category 2: One of several predicted diagnoses confirmed Category 3: Unexpected but treatable diagnosis made Category 4: Assessment of disease severity  Category 5: Non-diagnostic Category 6: Technical failure **This graph illustrates the diagnostic categories for the 196 biopsies with complete information that were reviewed**. Note that the values for percentage in the pie chart have been rounded off to whole numbers. 1, a biopsy that revealed the specific diagnosis predicted by the physician; 2, a biopsy that confirmed one of multiple possible diagnoses; 3, a biopsy that revealed disease that was different than the pre-biopsy prediction but which was conclusive and sufficient to determine appropriate treatment; 4, a biopsy done to determine the severity of an established disease; 5, a biopsy that was insufficient on its own to define prognosis and treatment; 6, a biopsy that was a technical failure with inability to obtain tissue.

Fifty-eight (29%) patients were classified as type 1, 28 (15%) were type 2, 27 (14%) were type 3, 59 (30%) were type 4 (Figure 2). Of the unsuccessful biopsies, 21 (10.7%) of the biopsies were inconclusive and further testing was required to confirm a diagnosis (type 5). In 3 of these cases, treatment was based upon the clinical findings rather than the histopathological diagnosis reported after the biopsy. In the remaining 18 cases, 5 patients were started on non-immunosuppressive, renoprotective treatment, 8 were followed without initiation of any disease-specific therapy, and 5 were lost to follow-up or transferred to internal medicine nephrology services for further care. Therefore, these biopsies were considered unhelpful. Finally, 3 (1.5%) biopsies failed to yield adequate tissue samples (type 6).

The diagnostic utility of the kidney biopsies based on the pre-biopsy diagnosis is summarized in Table 2. The percentage of diagnostically failed biopsies ranged from 0 to 33%. However, focusing on diagnostic entities that contained ≥ 10 patients, there were no significant differences in the rate of this problem for any specific clinical entity.

**Table 2:** Failure by Prebiopsy Diagnosis

Diagnoses			Failure	
**Diagnosis**	**N**	**%**	**N**	%
Systemic Lupus Erythematosus	66	34%	8	12%
Focal segmental glomerulosclerosis	59	30%	9	15%
IgA Nephropathy	32	16%	2	6%
Minimal Change Nephrotic Syndrome	17	9%	2	13%
Membranoproliferative Glomerulonephritis	16	8%	2	13%
Henoch Schoenlein Pupura Nephritis	12	6%	2	17%
Membranous Nephropathy	11	6%	2	18%
Acute Interstitial Nephritis	10	5%	2	20%
Hereditary nephritis (Alport/FTBMN)	10	5%	0	0%
Glomerulonephritis	7	4%	1	24%
Post Infectious Glomerulonephritis	7	4%	1	14%
Wegener granulomatosis	3	2%	1	33%
Miscellaneous	11	6%	1	9%

Abbreviation: FTMBN: familial thin basement membrane nephropathy

the number of pre-biopsy diagnoses exceeds the number of biopsies performed because each potential diagnosis that was considered as a clinical possibility before the procedure is listed in the Table.

## Discussion

In this review, useful biopsies were defined as those that definitively established the diagnosis and which provided solid guidance to the attending nephrologist on how to treat the patient. Of the 196 kidney biopsies done between 2000 and 2008 for which complete data were available, we determined that 172 (88%) were useful as defined above. In particular, of the 113 kidney biopsies that were done for diagnostic purposes (types 1, 2, 3) 85% were helpful, and all of the procedures done to assess severity were also deemed helpful. From a clinical perspective, categories 1–4 were all considered useful and could have been combined into one group. We divided them into four distinct subcategories to better characterize how nephrologists utilize the information content of a kidney biopsy in a productive manner.

In most circumstances, a renal biopsy is a semi-elective procedure, and technical failures can occur when the procedure fails to obtain adequate tissue. Of all the biopsies analyzed in this study, only 3 were technical failures. The fact that renal biopsies were not useful in 12.2% of cases needs to be considered in assessing the benefit of performing biopsies. In those cases, further testing is needed after the biopsy, and treatment has to be guided by clinical factors rather than the histopathological findings. In our study, we found the overall yield of uninformative biopsies was 12.2%, which is below the 20% threshold that we defined as an acceptable rate. This constitutes an acceptable risk/benefit ratio. Despite the potential risks involved, families can be reassured that a renal biopsy will be helpful in the great majority of patients who must undergo the procedure.

In judging this study, it is worth noting that there has been general uniformity in practice and staffing over the study period. One physician (HT) has been one of the primary nephrologists over the past 20 years and active throughout the 8-year study period and one pathologist (EV) interpreted all the biopsies from 2000 to 2007. It is reasonable to conclude that there was consistency in the use of kidney biopsies to predict, diagnose, and treat patients suspected of having kidney disease. The number of biopsies performed per year (Figure 1) showed no consistent trend, upward or downward, reinforcing the uniformity of the practice. We chose to focus on the years 2000–2008 in order to shed light on the utility of the kidney biopsy during a period that reflects current medical practice. Although it would have been optimal to have the interpretation of all biopsy reports reviewed by the independent observer, the four primary authors agreed in over 80% of cases. Moreover, the physician who performed the kidney biopsy was never involved in the retrospective assessment of diagnostic utility of the procedure. Despite the focused duration of the study and the single center nature of the review, a sufficient number of patients were identified that enabled conclusions to be drawn from our experience.

It is important to acknowledge that the study cannot definitively assess the utility of kidney biopsies performed to determine severity (type 4) in SLE patients. We assumed the results to be useful, excluding those in which a technical failure occurred, because the biopsies identified the WHO class of the disease in all cases and presumably guided treatment by the rheumatologists. This reflects the approach to patient management at Schneider Children's Hospital in which the care of children with SLE nephritis is delivered primarily by the rheumatologists unless the patients have specific issues such as hypertension, edema, or progress to end stage kidney disease. The utility of the biopsy may vary in those centers where nephrologists directly supervise the full care of patients with SLE. In addition, this study was limited to native kidney biopsies and did not address the value of transplant biopsies. Finally, we have not commented on complications of the procedure because we have reviewed our experience in a previous publication which confirms the overall safety of kidney biopsies performed using ultrasound localization [14].

Based upon clinical experience and perennial difficulties distinguishing between FSGS and MCNS clinically, we anticipated that kidney biopsies performed on these patients would have the highest non-diagnostic rate. Table 2 indicates, however, that the rate of unhelpful biopsies performed on FSGS and MCNS patients was not significantly different compared to biopsies performed for other conditions, and the maximum non-diagnostic rates for any of the major diseases was 10–20%. The entities with higher failure rates involved small numbers of patients, which precludes meaningful conclusions.

It is likely that nephrologists will always have to grapple with the fact that there will be a percentage of biopsies that fail to yield a diagnosis. Too small a percentage suggests too stringent requirements for performing a kidney biopsy. Conversely, a high percentage suggests that kidney biopsies are being performed for inadequate indications. The issue of an acceptable value will vary depending upon the urgency of the patient's clinical situation, the spectrum of clinical outcomes that might occur, and the availability of treatments that can materially alter the outcome of an anticipated disease entity. For example, pediatric surgeons recognize that not every child with abdominal pain who undergoes a laparotomy should have appendicitis because they realize it is better to occasionally find a normal abdomen rather than manage a ruptured appendix. Interestingly, a recent report in the surgical literature suggests that an acceptable negative appendectomy rate is 10–20%, in the range of our rate of negative kidney biopsies [15]. In view of the spectrum of kidney problems that mandated a biopsy, it may be difficult to define a single acceptable rate of non-diagnostic biopsies, and we offer our findings as a basis for future considerations on this issue.

In summary, we present our findings about the diagnostic value of a kidney biopsy in a varied group of pediatric patients. The overall rate of 12% seems to strike an appropriate balance between maximizing the application and minimizing the risk of this invasive procedure. Our findings may need to be reassessed as clinical information regarding specific disease changes and technical innovations are introduced in the performance of kidney biopsies and handling renal tissue specimens.

## Abbreviations

AIN: Acute interstitial nephritis; FSGS: Focal segmental glomerulosclerosis; GFRe: Estimated glomerular filtration rate; HSP: Henoch-Schonlein purpura; MCNS: Minimal change nephrotic syndrome; MPGN: Membranoproliferative glomerulonephritis; SLE: Systemic lupus erythematosus; U_P/C_: Urine protein:creatinine ratio; WHO: World Health Organization

## Competing interests

The authors declare that they have no competing interests.

## Authors' contributions

BS, AP, JR and FT retrieved patient data, collated the information and performed data analyses. RF, SV, CH, and BG assisted in patient identification, reviewed biopsy reports. EV and DC performed review of pathology data. BS, AP, and HT wrote the manuscript. HT conceived of the study and designed the retrospective review. All authors read and approved the final version of the manuscript

## Pre-publication history

The pre-publication history for this paper can be accessed here:

http://www.biomedcentral.com/1471-2369/10/11/prepub
